# Establishing core domains in suicide postvention: a Delphi study in Chile

**DOI:** 10.3389/fpsyt.2026.1840763

**Published:** 2026-06-12

**Authors:** Álvaro Jiménez-Molina, Thiare Barrera, Stefanella Costa-Cordella, Graciela Rojas, Vania Martínez, Paulina del Río, Marta Silva, Daniel Núñez

**Affiliations:** 1 Faculty of Psychology and Humanities, Universidad San Sebastián, Santiago, Chile; 2 Núcleo para Mejorar la Salud Mental de Adolescentes y Jóvenes (Imhay), Santiago, Chile; 3 Millenium Institute for Research in Depression and Personality (MIDAP), Santiago, Chile; 4 Department of Psychology, Universidad Academia de Humanismo Cristiano, Santiago, Chile; 5 Faculty of Psychology, Universidad Diego Portales, Santiago, Chile; 6 Hospital Clínico de la Universidad de Chile, Santiago, Chile; 7 CEMERA, Faculty of Medicine, Universidad de Chile, Santiago, Chile; 8 Fundación José Ignacio, Santiago, Chile; 9 Faculty of Psychology and Humanities, Universidad Austral de Chile, Valdivia, Chile; 10 Faculty of Psychology, Universidad de Talca, Talca, Chile

**Keywords:** Delphi study, healthcare professional training, Latin America, suicide postvention, suicide, suicide bereavement, suicide prevention

## Abstract

**Background:**

Postvention —structured support for individuals and communities exposed to or bereaved by suicide– is increasingly recognized as a core component of suicide prevention; yet standardized protocols and training frameworks remain scarce, particularly in low- and middle-income countries, leaving healthcare professionals without adequate guidance to respond effectively.

**Objective:**

To identify and reach expert consensus on the core domains of suicide postvention to inform a recommendation guide and a training program for healthcare professionals in Chile.

**Methods:**

We conducted a Delphi consensus study integrating academic, practice-based, and lived-experience expertise to identify and validate core domains for a postvention guide and online training program. Experts rated items on a five-point Likert scale, and consensus was defined *a priori* as ≥90% agreement on the two highest positive response options (“Important” + “Essential”) or an interquartile range (IQR) ≤1. A complementary online focus group with healthcare professionals from public health services in three Chilean regions assessed the materials’ perceived utility and implementation feasibility. Quantitative responses were analyzed with descriptive statistics; qualitative inputs were examined through content analysis (Delphi) and thematic analysis (focus group).

**Results:**

The Delphi panel comprised 28 experts (8 international researchers, 6 national researchers, 3 policymakers, 6 healthcare professionals, and 5 suicide-loss survivors), with 89% retention across two rounds. Consensus was reached on five core domains: (1) the impact of suicide and foundations of postvention; (2) preparedness and immediate response, including safe communication; (3) ongoing response, accompaniment, and recovery; (4) postvention in specific settings (education, workplace, and health services, including support for healthcare teams); and (5) long-term follow-up and prevention. In the complementary focus group (n=5 healthcare professionals), participants endorsed these domains and highlighted the need for early accompaniment within the first days post-suicide, integration of survivor testimonies, and concise asynchronous online modules adapted to the time constraints of public health professionals.

**Conclusions:**

Expert consensus on core suicide postvention domains and content provides the foundation for a recommendation guide and training framework designed for scalable implementation across Chile’s public health system and adaptable to other Latin American and resource-constrained contexts.

## Introduction

Suicide is a major public health problem worldwide, and exposure to suicide affects a substantial proportion of the population. Approximately one in twenty individuals is exposed to the suicide of someone they know each year, and one in five over their lifetime, with each death potentially affecting between 6 and 135 people (1, 2). Exposure to a suicide death has been associated with deterioration in physical and mental health and with disruptions in family and community dynamics (3–5). Moreover, the suicide death of a family member triples the likelihood of a subsequent suicide attempt, while the loss of a friend or acquaintance increases this risk 2.5-fold (6). Suicide postvention –a set of structured support actions for individuals, families, and communities affected by a suicide death (7)– therefore plays a key role in suicide prevention by reducing the risk of subsequent suicidal behavior among those exposed (6, 8).

Despite the growing body of research on suicide postvention over the past decade, evidence on its effectiveness remains limited (1, 9–12). The available evidence is also unevenly distributed across sociocultural contexts: most postvention studies and programs have been conducted in high-income countries of the Global North (12, 13). This leaves substantial gaps in regions such as Latin America, where culturally and contextually relevant guidance remains scarce. Addressing this imbalance requires incorporating a wider diversity of cultural and contextual perspectives, particularly from low- and middle-income settings where postvention infrastructure is still emerging.

Against this backdrop, the World Health Organization has urged countries to strengthen suicide postvention as a core component of comprehensive suicide prevention strategies (14). Chile illustrates the regional challenges of translating this mandate into practice. Although the National Suicide Prevention Program (NSPP) was established in 2013, it lacks standardized postvention protocols and formal training frameworks for healthcare professionals (15). Existing recommendations are largely restricted to media reporting. A 2023 evaluation of the NSPP identified the strengthening of postvention as a priority, calling for timely, culturally sensitive interventions to accompany suicide bereavement (16). Consistently, recent qualitative evidence shows that people bereaved by suicide encounter delayed responses and limited understanding within traditional health services, while healthcare professionals report insufficient training and orientations to provide adequate support (17).

Chile’s health system currently lacks the standardized protocols and training frameworks needed to ensure timely, coordinated, and high-quality postvention responses. Addressing this gap, the present study aimed to identify and reach expert consensus on the core domains of suicide postvention to inform the development of a recommendation guide and a training program for healthcare professionals in Chile, contributing to the strengthening of postvention capacity within the public health system.

## Methods

Given the limited evidence base in suicide postvention (12), this study adopted a qualitative design that combined a Delphi consensus process with a complementary focus group to identify and validate the core dimensions and contents of a postvention recommendation guide and training program. The Delphi method, widely used in health research and in suicide prevention to build expert agreement (18–21), was complemented by a focus group with healthcare professionals to incorporate practice-based, interactional insights (22). Together, these methods enhanced the validity and practical relevance of the findings, supporting the development of materials that are both expert-informed and contextually grounded (23).

**Figure 1:**
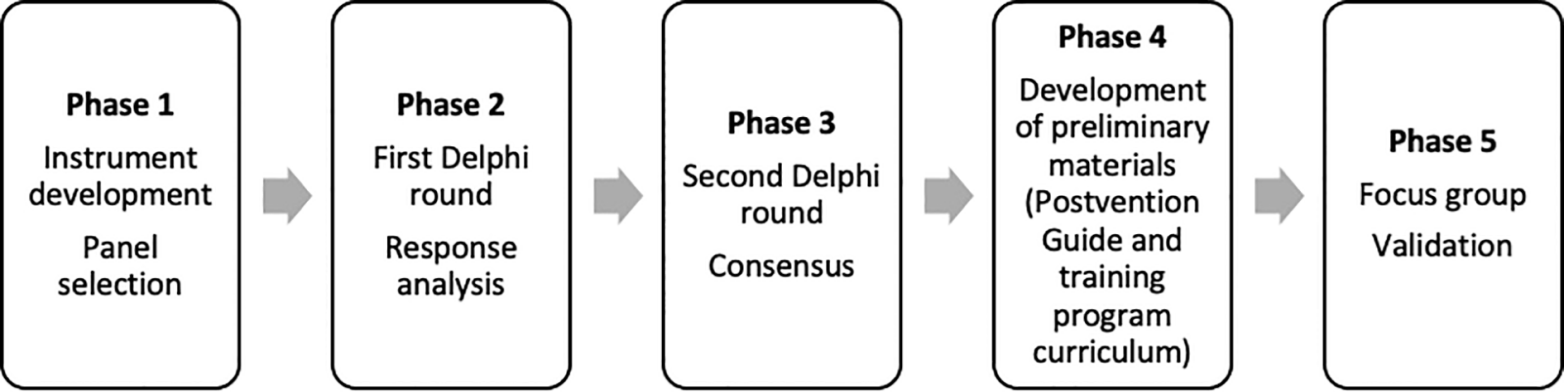
Phases of the information production process.

This study was conducted in five sequential phases (see **Figure 1**). Phase 1 focused on instrument development and expert panel selection. A multidisciplinary research team in mental health and suicide prevention (comprising three psychologists, two psychiatrists, one anthropologist, one sociologist, and one suicide-loss survivor) designed and refined the Delphi questionnaire and compiled a database of potential panelists to ensure disciplinary and geographic diversity. Initial Delphi statements were generated through a combined deductive and inductive approach. Deductively, the team drew on an integrative review of international postvention frameworks and guidelines (7, 10, 14, 24). Inductively, statements were informed by findings from previous qualitative studies of suicide-bereaved individuals and Chilean healthcare professionals (16, 17), which highlighted context-specific gaps and needs. An initial pool of candidate dimensions and contents was drafted by the research team, iteratively refined through internal review meetings, and piloted with two members of the team (one knowledge-based and one experience-based expert) before circulation, ensuring clarity and relevance.

Phase 2 comprised the first Delphi round. Experts evaluated an initial set of dimensions and contents for the recommendation guide and identified key components for the training program. The questionnaire combined two types of items: (1) closed-ended items rated on a five-point Likert scale (“I would not include it”, “Slightly important”, “Unsure – it depends”, “Important”, “Essential”) to evaluate and prioritize proposed contents for the recommendation guide; and (2) open-ended questions to identify key elements for the training program, including pedagogical objectives, content areas, and core competencies (knowledge, skills, and professional attitudes).

Open-ended responses from the second section of the questionnaire provided key input for the thematic categorization of priority content areas (e.g., critical competencies, intervention methodologies), which informed the design of the instrument used in the second Delphi round. Moreover, the qualitative comments guided the identification of essential aspects for developing both the recommendation guide and the training program. This process integrated both the quantitative validation and the qualitative insights of the expert panel.

Following established methodological recommendations (25, 18), quantitative and qualitative analyses were used to assess levels of agreement and identify key themes. Quantitative responses were analyzed using descriptive statistics (frequencies, percentages, medians, and interquartile ranges). Open-ended responses were analyzed through qualitative content analysis (26), using both deductive coding (anchored in the predefined dimensions) and inductive coding (allowing additional themes to emerge from the data). Two members of the research team independently coded the responses, and discrepancies were resolved through consensus discussions.

Consensus was operationalized using two complementary criteria: either ≥90% agreement on the two highest positive response options (“Important” + “Essential”) or an interquartile range (IQR) ≤ 1. The IQR is a robust measure of dispersion that is unaffected by extreme values; lower IQR values indicate greater concentration of responses (18). The 90% threshold was selected as a high-rigor criterion, consistent with prior Delphi studies in mental health that have adopted higher cut-offs when results are intended to inform clinical practice guidelines or training materials (19). Items meeting these criteria were accepted directly. Items falling below the consensus threshold were either excluded or revised in accordance with the experts’ qualitative comments and suggestions, and subsequently submitted to a second round of evaluation.

Phase 3 comprised a second Delphi round in which non-consensus items were re-evaluated following controlled feedback. Each participant received a summary of the aggregated panel responses alongside their own previous ratings, in line with standard Delphi procedures (19). The process concluded once stable consensus was achieved across the revised items, with no additional rounds required.

Phase 4 involved the development of the recommendation guide and training curriculum based on the Delphi results, followed by validation through a focus group with healthcare professionals. Delphi findings were translated into structured materials by mapping each consensus item to one of the five core domains and operationalizing it as a chapter section (for the guide) or a content module (for the training program). Qualitative inputs were used to refine content, clarify scope, and strengthen the cultural relevance of both materials. For example, expert comments on the non-linear nature of grief informed the adoption of process-oriented bereavement models emphasizing the singularity of each bereavement experience; and feedback on the need to support response teams beyond the immediate family led to the inclusion of a dedicated sub-section on disenfranchised grief in healthcare settings.

The focus group (Phase 5) was conducted online via Zoom, lasted approximately 90 minutes, and was facilitated by two members of the research team (a senior researcher and a research assistant trained in qualitative methods). Focus group participants received the materials developed in the preceding phases –including a draft version of the recommendation guide– three weeks prior to the session. A semi-structured guide was used to elicit feedback on (1) perceived strengths and weaknesses of the materials, (2) feasibility of implementation in public health services, (3) cultural and contextual relevance, and (4) preferred delivery formats. The session was audio-recorded, transcribed verbatim, and analyzed using thematic analysis (27). Themes were inductively derived and reviewed by the research team; findings were subsequently used to refine both materials, with particular attention to the development of concise, accessible formats –such as infographics– and the strengthening of references to coordination across levels of care and available public and private support resources.

### Participants

A non-probabilistic, purposive sampling strategy was employed to compose the participant groups. For the Delphi panel, four categories of experts were defined:

- Knowledge-based experts: National and international researchers in mental health and suicide prevention with extensive experience in prevention and/or postvention (operationalized as ≥10 years of research, intervention, or training activity in suicide prevention or postvention, and at least two peer-reviewed publication or technical report in the field).
- Practice-based experts: Health professionals (psychologists, physicians, nurses, among others) with experience working with individuals at risk of suicide and/or in postvention within public or private healthcare institutions (≥5 years of clinical or community-based practice).
- Policy experts: Decision-makers in the health sector responsible for the management and design of mental health policies and programs at regional or national levels.
- Experience-based experts: Suicide loss survivors (individuals bereaved by the suicide of a loved one and who have navigated the grieving process).

To constitute the Delphi panel, a minimum of 20 participants was established, consistent with recommendations for heterogeneous expert panels in Delphi research (18, 23). This minimum was also intended to ensure adequate representation across the four expert categories. Accordingly, 45 potential participants were identified and invited via email, anticipating the attrition rates typical of Delphi studies in specialized fields.

For the focus group, purposive sampling was used to recruit healthcare professionals with experience in public health services across the three Chilean regions where the training program will be piloted (Metropolitan, Maule, and Los Lagos). A target sample of 5–8 participants was established, in line with recommendations for focus groups in health research, where smaller groups facilitate in-depth discussion of complex topics (22). Invitations were extended to 12 professionals representing diverse disciplines — including occupational therapy, nursing, general medicine, and psychology — to ensure interdisciplinary breadth and to account for expected attrition. These professionals represent the primary end users of the recommendation guide and training program.

### Ethical approval and informed consent

This study was approved by the Research Ethics Committee of Universidad Diego Portales (Protocol No. 037-2023). All participants provided informed consent prior to their inclusion in the Delphi panel and focus group.

## Results

**Table 1** presents the distribution of invited participants and their actual participation in each round of the Delphi panel. In the first round, the response rate was 62%, yielding a final sample of 28 experts, of whom 20 were based in Chile and 8 were international. The international experts were from Australia (n=2), the United States (n=1), France (n=2), and the United Kingdom (n=3). Most national experts (n=18) resided in the Metropolitan Region. In terms of gender distribution, 15 male experts (53.6%) and 13 female experts (46.4%) participated. Among the group of health professionals (n=6), there were 3 psychologists, 2 psychiatrists, and 1 nurse.

**Table 1:** Delphi panel.

Experts	Contacted	Participants R1	Participants R2
International researchers	13	8	6
National researchers	10	6	6
Decision-makers	5	3	3
Healthcare professionals	10	6	6
Suicide loss survivors	7	5	4
Total	45	28	25

The results from the first round showed a high degree of consensus across most items, as reflected in the interquartile range and percentage agreement (see **Table 2**).

**Table 2:** Contents of the suicide postvention recommendation guide.

Item	Content	IQR	Agreement level
What is postvention?	Defines the general objectives of suicide postvention and presents strategies and actions to support individuals and communities.	0.75	100%
Immediate response	Provides practical guidance for early intervention following a suicide, including activation of support networks, strategies for offering emotional first aid, and organization of funerary rituals.	1	100%
Safe language	Presents guidelines for communicating with individuals affected by a suicide and addressing sensitive issues during bereavement while avoiding stigmatization.	1	100%
Media and social networks	Presents recommendations for managing information in social media and news outlets.	1	92%
Suicide bereavement	Offers tools to understand the impact of suicide and the bereavement process, including different conceptual models. Describes expected reactions and emotional responses following a suicide loss.	1	92%
Bereavement in children and adolescents	Addresses the needs of children and adolescents after a suicide, including their specific ways of expressing distress. Emphasizes the importance of age-appropriate and sensitive communication.	1	92%
Stigma	Addresses experiences of stigma associated with suicide among individuals, families, and communities.	1	92%
Complicated grief, trauma, and suicide risk	Describes the characteristics of complicated grief and potential traumatic effects affecting relatives, friends, and loved ones after a suicide, including increased suicide risk.	1	96%
Support for affected individuals	Identifies areas of support needed during bereavement and provides tools for self-care, strategies to assist others, and psychoeducational and emotional support resources.	1	92%
Postvention in educational settings	Presents guidelines for responding to a suicide death in school and higher-education settings at both individual and institutional levels.	0.75	96%
Postvention in workplace settings	Presents guidelines for addressing a suicide death in the workplace at both individual and organizational levels. Includes recommendations for planning the return-to-work process.	1	96%
Support for healthcare professionals	Describes the impact of a patient’s suicide on healthcare professionals and teams, and presents principles for institutional management. Addresses the concept of “disenfranchised grief” and promotes self-care and institutional strategies.	1	96%
Commemoration and everyday life	Offers guidance on coping with anniversaries and special dates related to the suicide.	1	96%
Postvention protocol	Presents general principles for developing postvention protocols in different settings (healthcare, educational, workplace). Includes a checklist of basic actions that should be implemented in suicide postvention interventions.	1	96%
Support services and resources	Presents available services and resources for individuals exposed or affected by a suicide death, as well as public and private suicide prevention services.	1	96%
Items below the consensus threshold			
Specific groups and communities	Presents guidelines for developing postvention strategies for specific groups, such as Mapuche populations or LGBTQ+ communities.	1	84%
Assessment and management of suicide risk	Presents practical tools for the initial evaluation of suicide risk and early identification of individuals most affected in the days following a suicide.	1	84%

Qualitative feedback underscored the importance of a flexible approach tailored to the different stages of bereavement, considering the diversity of experiences and personal meanings associated with this process. Participants emphasized the need to acknowledge that the impact of a suicide death extends beyond the family circle, affecting colleagues, peers, and response teams who may experience a sense of invisibility within support processes:

“It is essential to recognize that a suicide death can have impacts that go beyond grief or sorrow. The broader networks surrounding the deceased person –including colleagues, peers, professionals, and response teams (who may not have had a prior relationship with the deceased)– can experience shock, trauma, and associated symptoms, as well as anger and fear of being blamed or facing reprisals. Alongside these experiences, they may feel ignored or perceive themselves as less important compared to those who had a closer relationship with the deceased and are undergoing bereavement. [ … ] The use of the term ‘bereavement’ may lead many of these affected individuals to perceive that the available support is neither designed for them nor accessible” (International knowledge-based expert).

Policy experts contributed a systems-level perspective, emphasizing the need for postvention recommendations to be coordinated across the different levels of the healthcare network and aligned with existing national policy frameworks. Several participants also stressed the importance of keeping the recommendation guide focused exclusively on postvention, noting that suicide risk identification and management are complex processes that fall beyond its scope:

“I believe that [the recommendation guide] should focus on postvention, not on risk identification, which is a very complex and highly specific issue” (National knowledge-based expert).

“In my opinion, the module ‘Suicide risk assessment and management’ should be directed exclusively to mental health professionals. We should not assign this responsibility to other actors, whose role should be limited to detection and referral, and nothing more” (International knowledge-based expert).

Regarding intervention strategies, a general approach was recommended rather than one differentiating among specific groups. Participants pointed out, for example, that interventions aimed at Indigenous or LGBTQ+ communities require specialized knowledge of their specific contexts, and therefore, it would not be appropriate to include these aspects within the guide:

“I would not include postvention strategies aimed at Indigenous or LGBTQ+ populations, because grief is a general process, and addressing these groups adequately would require specific information on religiosity and spirituality in Indigenous communities and on LGBTQ+ issues, which would constitute a separate study in itself” (Practice-based expert).

Several participants emphasized that both the recommendation guide and the training program should highlight that grief is not a linear process but rather an experience through which individuals move non-sequentially across different stages. Some knowledge-based experts recommended updating traditional stage-based grief models toward more flexible process-oriented approaches that acknowledge the diversity of experiences and avoid rigid interpretations. Experience-based experts stressed the importance of allowing individuals to grieve at their own pace. One survivor, for example, emphasized avoiding prescriptive norms regarding how to cope with loss:

“Stage-based grief models are outdated and lack empirical support. Perhaps they should be updated, ideally toward process models and grief models that are not generic (since this case presents particular characteristics)” (National knowledge-based expert).

“[It is necessary] to make explicit that the stages of grief should be understood permissively –not as a consecutive process but rather as ‘stations’ we may or may not pass through, and sometimes revisit repeatedly in our emotional experience. Many survivors tend to believe they must necessarily ‘move forward’ through the stages or phases of grief” (Experience-based expert).

Some participants noted the importance of incorporating survivor testimonies into both the recommendation guide and the training program, to strengthen their experiential grounding and ensure that the perspectives of those with lived experience remained central to the final products.

Experience-based experts shared reflections on the actions or interventions they found helpful during their bereavement process. Beyond psychological or psychiatric support, they highlighted the importance of “being able to talk about the issue as many times as needed without being labeled as ‘stuck on it’” and “creating my own space through art to re-signify the loss.” One survivor emphasized the positive impact of reading the testimony of someone who had gone through a similar experience, while another underscored the value of peer-support groups and activism: “participating as an agent of change and support within my community.”

Based on these comments, a revised version of the questionnaire was developed in which some non-consensual items were reformulated, ambiguous concepts clarified, and technical specifications added when necessary. The second Delphi round, conducted two months after the first, maintained a high retention rate (89%), with 25 of the initial 28 experts participating. Once the predefined consensus criteria were met for both the recommendation guide and the training program, the Delphi process concluded without the need for additional rounds, ensuring the validity of the proposed content.

### Recommendation guide

As an outcome of expert consensus, the recommendation guide was structured into five core dimensions: (1) the impact of suicide and foundations of postvention; (2) preparedness and immediate response, including safe communication; (3) ongoing response, accompaniment, and recovery; (4) postvention in specific settings (education, workplace, and health services, including support for healthcare teams); and (5) long-term follow-up and prevention.

First, the dimension on the impact of suicide and the foundations of postvention emphasizes understanding the emotional and social consequences of suicide for individuals and communities, distinguishing between expected reactions and traumatic sequelae requiring specialized care, and clarifying key concepts and objectives of postvention.

Second, the first response dimension focuses on the initial days following a suicide, highlighting crisis management strategies such as psychological first aid, safe and responsible communication in media and social networks, the organization of funerary rituals, and the development of institutional postvention protocols.

Third, the support and recovery dimension address ongoing responses over the first 1–24 months, providing guidance on accompaniment during bereavement, self-care, psychoeducation, and the management of anniversaries, memorials, and the reorganization of everyday life after a suicide loss. Fourth, the specific contexts dimension includes tailored recommendations for educational, workplace, and healthcare settings, with particular attention to supporting healthcare professionals and clinical teams exposed to suicide deaths.

Finally, the implementation dimension outlines principles for ensuring preparedness through postvention protocols and provides information on available support services, national resources, and survivor testimony.

### Postvention training program

From the outset of the Delphi process, the training program was defined as targeting a broad range of healthcare professionals –including social workers, emergency personnel, general practitioners, psychologists, psychiatrists, and related practitioners– working across mental health services, family health centers, and emergency units within Chile’s public healthcare network. The program was designed for online, asynchronous delivery to enhance accessibility and feasibility. Within this framework, participants identified the core knowledge areas, skills, and professional attitudes required for effective suicide postvention. Across the Delphi rounds, 18 key content areas were initially proposed, of which 15 were validated through expert consensus in the second round (**Table 3**). The three items that did not reach the consensus threshold –the epidemiology of suicide, the components of the National Suicide Prevention Program (PNPS), and bereavement in specific populations (Indigenous and LGBTQ+ communities)– were excluded.

**Table 3:** Contents of the suicide postvention training program.

Item	Content	IQR	Agreement level
Definition and objectives of postvention	Presents the general objectives and guiding principles of suicide postvention.	1	100%
Impact of suicide on individuals, families, and communities	Describes common reactions and the potential impact of a suicide death on close contacts, families, and communities. Provides conceptual tools to understand suicide bereavement, emphasizing its heterogeneity.	1	96%
Stigma and trauma associated with a suicide death	Addresses stigma and the potential traumatic effects of suicide on loved ones.	1	96%
Impact of suicide on healthcare professionals	Addresses the potential impact of a patient’s suicide on healthcare professionals and provides guidance for institutional management (treatment documentation, communication with families, review of legal and ethical issues, etc.).	1	100%
Immediate response after a suicide (first days and weeks)	Provides practical guidance for information management, activation of support networks, assistance with post-suicide administrative tasks, and organization of funerary rituals.	0.75	100%
Crisis intervention/Emotional first response	Presents strategies for crisis intervention and emotional support during acute distress.	1	100%
Initial assessment and management of suicide risk	Presents practical tools for the detection, assessment, and initial management of suicide risk.	1	96%
Communication and safe language	Presents tools for effective communication with adults affected by suicide, including practical examples for addressing sensitive topics during bereavement.	1	100%
Emotional containment and expression	Addresses tools for providing emotional containment to individuals affected by a suicide death.	0.75	100%
Bereavement in children and adolescents	Provides tools to communicate with children and adolescents about suicide using age-appropriate and sensitive language, and strategies to support them in processing the loss.	1	96%
Ongoing response	Provides general guidelines for accompanying the bereavement process for up to two years after the suicide, identifying key areas of support.	0.75	100%
Psychoeducation	Offers general guidelines for psychoeducation for individuals exposed or affected by a suicide death, as well as self-care tools.	1	100%
Counseling and psychotherapeutic support	Provides general principles for counseling and psychotherapeutic work with people bereaved by suicide.	1	92%
Peer-support groups	Presents the objectives of mutual support groups and offers general guidance for their implementation under the supervision of healthcare professionals.	1	92%
Support services and resources	Presents information on available support services and resources for individuals exposed or affected by a suicide death, as well as public and private suicide prevention services.	1	100%
Items below the consensus threshold			
Epidemiology of suicide	Presents the main epidemiological indicators associated with suicidal behavior in Chile.	0.75	80%
Components of the National Suicide Prevention Program	Presents the components of the PNPS and their relevance to postvention.	0.75	80%
Bereavement experiences in specific groups	Presents guidance for developing postvention strategies for Mapuche populations and LGBTQ+ communities.	1	80%

Qualitative feedback emphasized the importance of communication and emotional support skills, particularly active listening, empathic communication, and the capacity to discuss suicide openly. Participants underscored the central role of emotional containment during crises and the importance of supportive accompaniment throughout the grieving process, emphasizing the relational and human dimensions of postvention:

“During the training process, it is important to emphasize that postvention interventions are processes in which the human and relational aspects are far more important than the technical ones. It is therefore essential to avoid allowing evaluation or intervention processes to become mechanical and/or impersonal” (Practice-based expert).

Some participants stressed the need for caution with peer-support groups, highlighting the importance of professional supervision and clearly defined objectives to ensure safety and effectiveness:

“One issue that raises concerns for me is related to support groups, because they could be counterproductive. The key would be to promote spaces or groups that are professionally supervised, have clear objectives, and take care to avoid unhelpful or harmful dynamics” (National knowledge-based expert).

Participants also highlighted the importance of self-care among those providing support, including the identification of burnout risks, management of emotional load, and regulation of personal emotional responses when addressing sensitive topics:

“I think it’s important that health professionals have space within the training to reflect on their own understanding, emotions, and responses to suicide. What implicit biases are they bringing into the conversation about this behavior?” (International knowledge-based expert).

Experts further observed that the training process itself can pose emotional challenges for health professionals. Among the main risks identified were the reactivation of memories of prior suicide-related experiences or losses, increased feelings of hopelessness, and anxiety about working with suicidal individuals. Therefore, participants emphasized the need to allow trainees to withdraw or pause the training if needed, as well as to adopt a reflective approach to help mitigate the emotional impact of the learning process.

As shown in **Table 3**, the training program encompasses core dimensions of immediate intervention, long-term accompaniment, and strengthening of support networks. In addition to establishing the conceptual foundations of postvention –its objectives and guiding principles– the program includes an immediate intervention phase (first days and weeks), which covers knowledge and skills for managing information, activating support networks, organizing funerary rituals, and providing psychological first aid, alongside specific crisis intervention strategies.

In the continuous accompaniment phase (up to two years post-suicide), the program addresses the emotional and social impact on individuals, families, and communities, emphasizing appropriate approaches to grief in children and adolescents, managing stigma, and identifying potential traumatic sequelae. It also explores the repercussions of suicide among healthcare professionals, addressing institutional case management and the promotion of self-care practices.

The training program incorporates support and prevention strategies, including tools for safe and respectful communication, emotional expression and regulation during grief, psychoeducation, and basic competencies in suicide risk assessment and management. While the expert panel emphasized that the recommendation guide should focus exclusively on postvention, consensus supported the inclusion of suicide risk assessment skills within the training program as a complementary capacity aimed at early identification, safe referral, and coordination within the healthcare system, rather than comprehensive clinical risk management. The program also establishes principles for counseling and psychotherapeutic accompaniment of survivors, provides guidance for the operation of peer-support groups, and offers information on specialized services and suicide prevention helplines.

The training program’s content can be organized into three strategic dimensions, designed to guide comprehensive interventions (see **Table 4**).

**Table 4:** Dimensions of the suicide postvention training program.

Dimension	Objective	Key Components
Impact of suicide and foundations of postvention	To identify the objectives of postvention and understand the emotional and social impact of suicide	• General objectives of postvention. • Impact of suicide on individuals and communities (emotional consequences, common reactions). • Associated stigma and its effect on bereavement. • Potential traumatic effects of suicide. • Impact of suicide on healthcare professionals.
Immediate response to a suicide death	To establish intervention guidelines for the first hours and days after a suicide	• Ethical management of information (communication with family members, schools, and communities). • Organization of funerary rituals with a preventive approach. • Activation of support networks and psychological first aid. • Initial assessment of suicide risk for early detection and safe referral. • Tools for crisis intervention and emotional support. • Empathic communication and non-pathologizing language.
Support for suicide bereavement processes	To accompany the bereavement process, sustain recovery, and prevent relapses	• Addressing social stigma and traumatic sequelae. • Psychoeducation and self-care techniques for those affected. • Differentiated strategies for children, adolescents, and adults. • Support for healthcare professionals. • Principles for counseling and referral to specialized psychotherapy (complicated grief, traumatic sequelae). • Principles for facilitating mutual support groups under expert supervision. • Access to community resources (helplines, support services for survivors).

### Focus group

The focus group comprised five healthcare professionals from three regions of Chile (two from Los Lagos, two from the Metropolitan Region, and one from Maule) representing occupational therapy, nursing, and psychology.

Participants valued the innovative nature of the materials and highlighted their contribution to addressing the lack of postvention training in Chile, emphasizing the contextualized content, clear language, broad professional scope, and inclusion of testimonies from individuals bereaved by suicide. At the same time, they identified operational constraints within health services—particularly limited time and delayed responses in the absence of clear guidelines—and recommended prioritizing concise, easily accessible formats, such as infographics or pocket guides, to support early postvention actions, especially during the first days following a suicide. They also stressed the need for coordination across different levels of the health system and positively assessed the inclusion of psychological first aid, despite its limited current use within a postvention framework.

Participants described the training program as a highly relevant and feasible tool, particularly due to its online, asynchronous format, which accommodates high workloads and territorial inequalities in access to training. They noted the importance of specifying the total instructional hours to facilitate accreditation within the Chilean health system and professional career pathways. While they appreciated the consideration of different practice contexts, participants suggested further development of content addressing specific populations—such as LGBTQ+ individuals and Indigenous peoples—to enhance cultural relevance and applicability across diverse settings. Overall, the focus group regarded both the recommendation guide and the training program as essential resources for strengthening postvention capacity across the public health network, underscoring the importance of concise, user-friendly formats that can be applied beyond specialized services.

## Discussion

Given the absence of systematic and culturally attuned suicide postvention strategies in Chile (17) and the limited global evidence on effective postvention models (1, 10, 13), particularly in Latin America (12), this study aimed to identify and reach expert consensus on the core domains of suicide postvention to inform the development of a recommendation guide and a training program for healthcare professionals.

By integrating empirical evidence, expert consensus, and lived-experience perspectives through a multidisciplinary Delphi panel, this study establishes a framework of interdependent postvention domains that reflects both international priorities (1, 28, 29) and the specific challenges of the Chilean public health system (16). Beyond its national relevance, the study addresses global gaps in postvention research: by generating a context-sensitive but internationally aligned framework from Latin America, this work offers a transferable example of how Delphi consensus methods can bridge international evidence and local implementation needs.

The five validated core domains align closely with established postvention frameworks while extending them in two ways. The dimensions of immediate response, ongoing accompaniment, and postvention in specific settings are broadly consistent with systematic reviews (10) and international guidelines (7, 14), which highlight crisis-phase intervention, longer-term support, and setting-specific recommendations as cornerstones of postvention. However, the framework derived from this study extends previous models by explicitly integrating support for healthcare professionals exposed to patient suicide as a dedicated domain rather than a peripheral concern, in line with emerging evidence on clinician bereavement and disenfranchised grief (30); and emphasizing the articulation between immediate response and long-term follow-up as a single integrated continuum, rather than as separate intervention streams.

The high degree of consensus achieved across the first Delphi round merits careful interpretation. Rather than indicating that the proposed domains were uncontroversial, this convergence likely reflects three interrelated dynamics: a widely shared diagnosis that postvention has been systematically underdeveloped, with broad agreement on the priorities required to address this gap; the resonance of the initial dimensions –anchored in both international evidence and prior Chilean empirical work– with stakeholders’ experiential and clinical knowledge; and the small community of postvention experts in Chile, who shared similar readings of contextual needs. This pattern is consistent with Delphi studies in narrowly defined specialty areas, where small but highly aligned expert communities tend to reach consensus more rapidly (19).

The items that did not reach the consensus threshold are equally informative. Across both materials, these concerned culturally specific strategies for Indigenous and LGBTQ+ populations, suicide risk assessment and management, local epidemiology, and national policy infrastructure. Qualitative comments suggest that the exclusion of culturally specific content reflects expert caution about scope rather than a dismissal of its importance: panelists viewed culturally sensitive postvention as requiring dedicated community involvement that exceeded a general guide. The contested status of suicide risk assessment reflects an ongoing tension regarding the appropriate boundaries of postvention, which the panel resolved by retaining it as a complementary competency in the training program –oriented toward detection and referral– while keeping it outside the recommendation guide. Items related to epidemiology and national policy were likely viewed as contextual background rather than core postvention competencies.

The four stakeholder groups contributed complementary perspectives that collectively strengthened the validity and practical relevance of the resulting framework. Knowledge-based experts grounded the discussion in current evidence, advocating for process-oriented bereavement models and clearer boundaries between postvention and suicide risk management. Practice-based experts foregrounded implementation realities, emphasizing relational intervention approaches. Policy experts highlighted the importance of coordinating postvention recommendations across levels of care. Experience-based experts brought irreplaceable insights into lived bereavement, underscoring non-prescriptive grief trajectories and the role of testimonies in re-signifying loss.

The recommendation guide and training program together address key postvention priorities –including stigma, traumatic responses, and the heterogeneity of suicide bereavement– while promoting flexible, process-oriented understandings of grief and extending postvention efforts beyond immediate family to include peers and first-response teams. The training program further responds to well-documented gaps in clinicians’ preparedness (30, 31) by incorporating suicide risk assessment, counseling accompaniment, and dedicated content on the impact of suicide on healthcare professionals. Focus group findings support the relevance of both materials while highlighting structural constraints –limited training time, the need for concise formats, and the importance of clear guidance during the immediate post-suicide period– and underscore a central challenge in postvention guideline development: balancing generalizable, evidence-based recommendations with the context-specific needs of everyday practice. These findings align with international evidence on sustained bereavement support, stigma reduction, and survivor-informed approaches (10, 21, 29, 31), reinforcing the urgent need for specialized training in this area (10, 30), and underscoring that postvention should be understood not as a secondary component of suicide prevention, but as a core capacity requiring standardized guidance, dedicated training, and institutional preparedness.

### Strengths and limitations

A major strength of this study lies in its integration of transdisciplinary perspectives, bringing together researchers, healthcare professionals, policy decision-makers, and suicide survivors, as well as national and international experts and practitioners from three regions of Chile. This diversity enhances the ecological validity and contextual relevance of the proposed postvention framework. The high level of consensus achieved across Delphi rounds further strengthens the study, providing a robust foundation for refining and implementing the recommendation guide and training program. The study’s design reflects its cultural and institutional anchoring: the questionnaire was developed by a Chilean research team in dialogue with local survivors and healthcare professionals; the panel combined Chilean and international experts to balance global evidence with contextual fit; and the focus group included practitioners from three regions representing the public health system where the materials will be implemented. The resulting framework is therefore not a generic application of international guidelines, but a context-specific synthesis oriented to the Chilean public health setting while remaining transferable to similar contexts where postvention is emerging.

Nonetheless, several limitations should be acknowledged. The relatively small pool of postvention experts in Chile and internationally constrained the size of the Delphi panel, and the concentration of national experts in the Metropolitan Region may have introduced geographic bias in the prioritization of certain themes. Additionally, the sample size within each stakeholder category was insufficient for stratified quantitative comparisons across groups; differences were therefore examined qualitatively. Future research should seek broader regional representation and greater involvement of community-based organizations to enhance the cultural relevance and applicability of postvention resources.

### Future directions

Based on these findings, a final version of the recommendation guide and the postvention training program will be developed. The program’s acceptability, feasibility, and perceived effectiveness will be evaluated among healthcare professionals from three regions of the country (Metropolitan, Maule, and Los Lagos). Findings from this evaluation will inform the final version of the program, with the aim of helping to address the existing gap in knowledge and training in suicide postvention.

## Conclusion

This study provides a contextually grounded foundation for a suicide postvention recommendation guide and training program, establishing standards of care for individuals, families, and communities exposed to or affected by suicide (from immediate crisis response to long-term accompaniment). These resources represent a critical step toward closing the postvention gap in Chile, with the potential to improve the quality, safety, and consistency of support across the public health system. By bridging academic research and public health practice through expert consensus, the study offers a basis for advancing policies, guidelines, and training initiatives that strengthen comprehensive suicide prevention efforts, while providing a transferable example for contexts where postvention infrastructure is still emerging.

## Data Availability

The raw data supporting the conclusions of this article will be made available by the authors, without undue reservation.
